# Immunoexpression Analysis and Prognostic Value of BLCAP in Breast Cancer

**DOI:** 10.1371/journal.pone.0045967

**Published:** 2012-09-25

**Authors:** Irina Gromova, Pavel Gromov, Niels Kroman, Vera Timmermans Wielenga, Ronald Simon, Guido Sauter, José M. A. Moreira

**Affiliations:** 1 Cancer Proteomics, Genome Integrity Unit, Danish Cancer Society Research Center, Copenhagen, Denmark; 2 Danish Centre for Translational Breast Cancer Research (DCTB), Copenhagen, Denmark; 3 Department of Breast Surgery, Copenhagen University Hospital, Copenhagen, Denmark; 4 Department of Pathology, the Centre of Diagnostic Investigations, Copenhagen University Hospital, Copenhagen, Denmark; 5 Department of Pathology, Diagnostic Center, University Medical Center Hamburg-Eppendorf, Hamburg, Germany; 6 Section of Pathobiology and Sino-Danish Breast Cancer Research Centre, Department of Veterinary Disease Biology, Faculty of Health and Medical Sciences, University of Copenhagen, Copenhagen, Denmark; Innsbruck Medical University, Austria

## Abstract

Bladder Cancer Associated Protein (BLCAP, formerly Bc10), was identified by our laboratory as being down-regulated in bladder cancer with progression. *BLCAP* is ubiquitously expressed in different tissues, and several studies have found differential expression of BLCAP in various cancer types, such as cervical and renal cancer, as well as human tongue carcinoma and osteosarcoma. Here we report the first study of the expression patterns of BLCAP in breast tissue. We analyzed by immunohistochemistry tissue sections of normal and malignant specimens collected from 123 clinical high-risk breast cancer patients within the Danish Center for Translational Breast Cancer Research (DCTB) prospective study dataset. The staining pattern, the distribution of the immunostaining, and its intensity were studied in detail. We observed weak immunoreactivity for BLCAP in mammary epithelial cells, almost exclusively localizing to the cytoplasm and found that levels of expression of BLCAP were generally higher in malignant cells as compared to normal cells. Quantitative IHC analysis of BLCAP expression in breast tissues confirmed this differential BLCAP expression in tumor cells, and we could establish, in a 62-patient sample matched cohort, that immunostaining intensity for BLCAP was increased in tumors relative to normal tissue, in more than 45% of the cases examined, indicating that BLCAP may be of value as a marker for breast cancer. We also analyzed BLCAP expression and prognostic value using a set of tissue microarrays comprising an independent cohort of 2,197 breast cancer patients for which we had follow-up clinical information.

## Introduction

Our laboratory has carried out a number of systematic and comprehensive proteomic studies to identify protein markers that may form the basis for improved diagnosis and prognosis as well as identify novel potential targets for therapeutics of cancer patients. Accordingly, we initiated two large proteomic projects focused on bladder and breast cancer, respectively [1]–[5]. The two initiatives have been implemented in a staggered fashion with the bladder cancer preceding the breast program, such that data generated in the first project could supplement and facilitate the discovery process in the second project. Our strategy to search for biomarkers relies on two-dimensional polyacrylamide gel electrophoresis (2D PAGE)-based differential proteomic profiling of matched normal and neoplastic fresh tissue. To date we have examined the protein expression profiles of thousands of tissue samples, using gel-based proteomics, and we have identified a number of potentially useful biomarkers for urinary bladder cancer [6]–[13], specific markers that can distinguish subtypes of breast cancer [14]–[16], as well as candidate serological biomarkers for breast cancer [17], [18]. One of the biomarkers we discovered, Bladder Cancer Associated Protein (BLCAP), was originally identified by our laboratory in a small study comprising 30 urothelial carcinomas (UCs) where we showed that loss of BLCAP mRNA expression correlates with the invasive potential of UCs [11]. To provide stronger evidence for BLCAP usefulness as a biomarker, we followed up on our initial observation and recently reported a validation study examining the protein expression pattern of BLCAP in a very large number of well characterized bladder samples with long-term clinical follow-up [13]. Our results, although confirming in a very large number of samples (over 1800 bladder cancer patients were examined) the original observation that BLCAP expression is lost with tumor progression, also showed that BLCAP is over-expressed in approximately 20% of the cases examined, and that strong nuclear expression is linked with poor disease outcome, suggesting that BLACP may also have prognostic value. Further studies by other laboratories have all found downregulation of *BLCAP* gene expression in the various cancer types examined, such as cervical [19], and renal cancer [20], as well as human tongue carcinoma [21], human osteosarcoma [22], and α-radiation-induced rat osteosarcoma [23]. In addition, over-expression of BLCAP in human TC-135 Ewing’s sarcoma cells and HeLa cervical cancer cells [24], [25] resulted in cell growth inhibition and induced apoptosis, indicating a role for this protein in the regulation of tumor cell proliferation and survival. Collectively these observations suggested that BLCAP may play a part in cellular carcinogenesis. However, the precise biological function of BLCAP is still largely unknown. BLCAP is a small (87aa), evolutionary conserved protein, with a high degree of similarity to orthologs, but no significant homology to any other known protein. *BLCAP* is ubiquitously expressed in different tissues and is a target for RNA editing via adenosine to inosine (A-to-I) RNA editing catalyzed by members of the double-stranded RNA (dsRNA)-specific Adenosine Deaminase Acting on RNA (ADAR) family [26]–[29].

Presently, there isn’t a single molecular marker able to detect early stage breast tissue changes or predict with accuracy the biological potential of breast lesions [30]–[32]. Moreover, for almost all cancer types, the protein biomarkers that have been identified to date do not possess the sensitivity and/or specificity required to have clinical utility individually. As a result, molecular cancer diagnostics is moving towards a multiplex-marker setting or even pattern diagnostics, in which protein signatures may be eventually employed in a clinical setting as a diagnostic test [33]–[39]. Accordingly, a multiple biomarker approach has been used by different groups to increase the diagnostic or prognostic value of markers. We have explored a similar approach, examining the markers we identified by proteomic analysis of cancer tissue samples, validating them in independent datasets and characterizing them in greater detail, with the hypothesis that candidate protein biomarkers that are not clinically useful individually, ultimately may have value within a panel of protein biomarkers.

Following the conceptual biomarker development phases proposed by the Early Detection Research Network [40], we have now started post-discovery validation studies for some of the most promising candidate proteins we have identified in our proteomics programs. Consequently, and given the potential usefulness of BLCAP as prognostic biomarker in human bladder cancer, the general differential expression of BLCAP in the many cancer types examined, and the potential role for BLCAP in cellular malignancy, we investigated the expression and distribution pattern of BLCAP in breast tissue, normal as well as malignant. In order to thoroughly characterize BLCAP protein expression and cellular localization patterns in normal breast and mammary carcinomas we used two independent sets of samples from different patient cohorts; a reference set consisting of specimens (formalin-fixed as well as frozen biopsies) from 123 patients with primary breast cancer collected within the framework of the Danish Centre for Translational Breast Cancer Research (DCTB), and an independent, large, validation dataset (2,197 samples) of clinically annotated breast cancer specimens [41]. We show here that BLCAP protein overexpression defines a subset of breast carcinomas with unfavorable outcome.

## Results

### Patterns of BLCAP Expression in Breast Tissue

In order to characterize the expression and cellular localization patterns of potential protein markers, our group typically utilizes an investigational framework consisting of a combination of 2D PAGE-based analysis of fresh tumors and matched benign tissue specimens, complemented by western blotting and immunohistological methods. In this manner, protein spots, surmised to be the marker(s) of interest, can be unequivocally identified by mass spectrometry (MS)-based analysis. Two-dimensional gel image spot matching and MS-based protein identification can thus be effectively combined with immunohistochemistry (IHC), addressing one of the major caveats of the latter technology, analyte specificity, and providing an orthologous cross-validation approach that delivers conclusive evidence for the differential expression of a marker in tumor tissues as compared to non-malignant samples. In the case of BLCAP, however, expression was, in all instances tested, below the detection limit of 2D silver stained gels, and neither urothelial carcinomas (13) nor breast tumors (Fig. S1) displayed BLCAP as a evident spot on 2D PAGE gels (all spots present in and around the MW and pI gel region of interest were tested by mass spectrometry).

**Figure 1:**
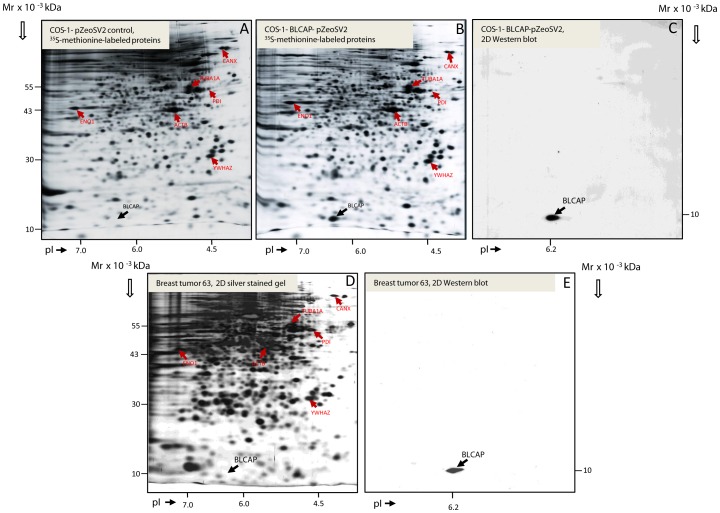
2D PAGE and 2D Western blot analysis of BLCAP protein spot patterns. (A) COS-1 cells transfected with pZeoSV2 empty vector and labeled with ^35^S-methionine. (B) COS-1 cells transfected with pZeoSV2– BLCAP overexpressing construct and labeled with ^35^S-methionine. Radioactive metabolic labeling (^35^S-methionine) of COS-1 cells was used to ensure the highest detection sensitivity. (C) 2D Western blot of COS-1 cells transfected with pZeoSV2– BLCAP construct detected with anti-BLCAP antibody (10 sec film exposure). (D) 2D gel of proteins from breast tumor 63 stained with silver. (E) 2D Western blot of protein lysate from breast tumor 63 (see D) reacted with anti-BLCAP antibody (1 min film exposure). The positions of the BLCAP protein in the 2D-PAGE gels and corresponding 2D Western blots, are indicated by black arrows. The positions of several reference proteins are indicated by red arrows: ACTB – beta actin; ENO1 -alpha enolase 1; CANX – calnexin; PDI - Protein disulfide-isomerase; TUBA1A - tubulin alpha-1A chain; YWHAZ - 14-3-3 protein zeta/delta. The identity of all reference spots were confirmed by MS analysis.

We have previously described a peptide-specific anti-BLCAP antibody, raised against a unique C-terminal peptide of BLCAP (aa 74–87) [13]. Antibody specificity evaluated by 2D-Western blot and IHC analysis showed that this antibody was specific towards the BLCAP protein in urinary bladder tissue [13]. To evaluate the potential of BLCAP as possible breast cancer biomarker and prognostic factor, we investigated the expression and distribution pattern of BLCAP in breast tissue, normal as well as malignant, using this antibody. To address the issue of antibody cross-reactivity and rule out a potential breast tissue-specific confounder of the analyses, we performed a comparative matching of corresponding spots on 2D Western blot of breast tissue lysates and extracts from COS-1 cells overexpressing BLCAP. 2D gel images of total protein extracts from breast biopsies were compared with 2D images of cell extracts from COS-1 cells transfected with the pZeoSV2-BLCAP expression construct (Figs. 1A, B and D). As shown in Fig. 1, transient transfection of COS-1 cells with the pZeoSV2-BLCAP construct yielded a novel protein spot of MW 10 kDa and pI 6.2 (compare Fig. 1A with 1B, black arrows), which exactly corresponds to the theoretical MW and pI values for BLCAP. Mass spectrometry analysis confirmed the identity of the spot (Fig. 1B, black arrow) as BLCAP (Fig. S2). 2D Western blots of BLCAP-overexpressing COS1 cells and a breast tumor sample, T63, were reacted with the BLCAP C-term antibody (Figs. 1C and E, respectively), which resulted in an analogous pattern in both samples. Comparative matching of the 2D PAGE images, performed by the PDQUEST software (v.8.0.1, Bio-Rad) using several reference proteins (Figs. 1B and D, respectively, red arrows), showed an exact overlapping of the position of the protein spots detected by the BLCAP antibody in breast tumor samples and BLCAP overexpressing COS-1 cells.

**Figure 2:**
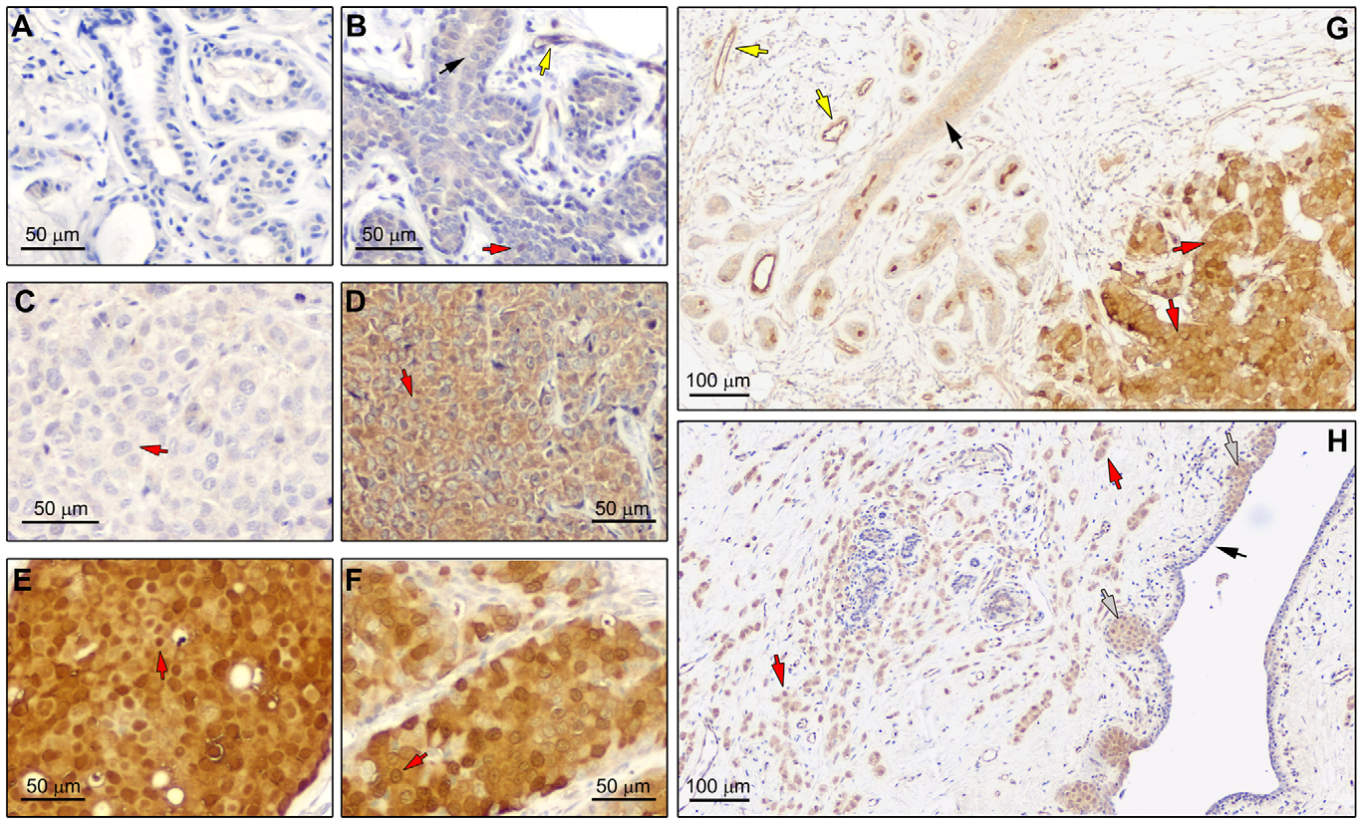
Immunohistochemical expression analysis of BLCAP in FFPE breast tissue samples. (A) No immunostaining was observed in a normal breast tissue section reacted with BLCAP antibody preincubated with immunizing peptide. (B) Immunohistochemical staining of BLCAP protein in a normal breast tissue sample demonstrated the presence of the BLCAP antigen in luminal epithelial cells with weak cytoplasmic expression (black arrow). Marked nuclear expression was also observed occasionally (red arrow). Yellow arrow points to a vessel with moderate immunoreactivity for BLCAP. (C) In a few cases IHC analysis of tumor samples showed that BLCAP was expressed in tumor cells with weak cytoplasmic expression (red arrow). (D) Most cases showed moderate to strong cytoplasmic expression with no detectable nuclear presence (red arrow) but in some cases (E) we could observe strong nuclear expression of BLCAP (red arrow). (F) In a few cases, samples were heterogenous with some cells showing distinct perinuclear immunoreactivity for BLCAP (red arrow). (G) Malignant cells showed stronger immunoreactivity (red arrows) than adjacent normal-looking ducts (black arrow), demonstrating up-regulation of this protein in tumor cells. Yellow arrows point to vessels with strong immunoreactivity for BLCAP. (H) We also observed up-regulation of BLCAP in early lesions where lobular carcinoma in situ cells showed overexpression of this protein (grey arrows) in relation to normal adjacent areas (black arrow), and at levels comparable to invasive carcinoma cells (red arrow).

The specificity of the anti-BLCAP antibody for IHC analysis of breast tissue was further examined by staining tissue sections with antibody pre-incubated with the corresponding immunizing peptide (Fig. 2). As shown in Fig. 2A, exposure of the antibody to the immunizing peptide prior to IHC effectively blocked immunostaining of normal breast tissue sections, confirming the specificity of the antibody towards BLCAP in mammary tissue. These data clearly demonstrate that the antibody reacts exclusively with BLCAP in breast tumor samples, thus ruling out any potential tissue-specific cross-reactivity confounder, and allowing us to use it in subsequent analyses.

IHC staining of normal breast specimens with the BLCAP antibody showed that the BLCAP antigen is present in mammary epithelial cells with weak to moderate cytoplasmatic staining (Fig. 2B, black arrow). Expression of BLCAP was not restricted to epithelial cells as we also detected immunostaining of vessels (Fig. 2B, yellow arrow), corroborating our previously reported observation that BLCAP is expressed by endothelial cells (ECs) in the vasculature [13]. Interestingly, in contrast to what we had previously observed for normal bladder tissue [13], where BLCAP shows strong interspersed nuclear expression in urothelial cells, in normal mammary tissue we only observed prominent nuclear expression of BLCAP in a few occasional breast epithelial cells (Fig. 2B, red arrow). In conclusion, we observed weak immunoreactivity for BLCAP in normal epithelial cells, almost exclusively localizing to the cytoplasm (Figs. 2B and G, black arrows), with moderate to strong expression in vessels (Figs. 2B and G, yellow arrows).

To study changes in the pattern of BLCAP expression that might take place during progression from normal mammary epithelium to breast cancer, we analyzed a reference set of specimens collected from 123 patients that are part of the breast proteomics initiative within DCTB (Table 1). We obtained interpretable staining results for BLCAP in a total of 101 tumor samples; the reason for failure was insufficient tumor cells available for scoring. IHC analysis of formalin-fixed paraffin-embedded (FFPE) sections from tumor specimens showed a wide variation in the patterns of BLCAP expression in breast cancer. We encountered samples with weak or no detectable immunoreactivity for BLCAP (Fig. 2C), although these constituted only a minor part (8%; 8 out of 101 tumor samples) of the total number of samples. The majority of the breast tumor samples examined (73%; 74 out of 101 tumor samples) was characterized by moderate to strong cytoplasmic staining of malignant cells with no discrete nuclear immunoreactivity (Fig. 2D). Specimens with tumor cells displaying marked nuclear immunoreactivity for BLCAP were also observed in 19% (19 out of 101; with 1% or more cells having nuclear staining) of the tumor specimens examined (Fig. 2E, red arrow). In addition, occasionally we also observed distinct perinuclear localization of BLCAP (Fig. 2F, red arrow).

**Table 1:** Clinical characteristics of DCTB tissue specimens.

Number of patients	123
Median age [range (years)]	62 (27–99)
Median tumor size [range (mm)]	28 (10–110)
Histologic type [*n* (%)]	
Ductal	100 (81)
Lobular	19 (16)
Other	4 (3)
Histologic grade [*n* (%)]	
Grade 1	27 (22)
Grade 2	57 (46)
Grade 3	37 (30)
Unknown	2 (2)
Lymph node status [*n* (%)]	
Positive	94 (76)
Negative	26 (21)
Unknown	3 (24)
Estrogen status [*n* (%)]	
Positive	99 (80)
Negative	24 (20)
Progesterone status [*n* (%)]	
Positive	75 (61)
Negative	48 (39)
HER-2/neu status [*n* (%)]	
0	25 (20)
1	35 (28)
2	34 (28)
3	29 (24)

The substantial number of specimens with strong immunoreactivity for BLCAP, and increased relative to what we had observed in normal specimens, suggested that this protein was overexpressed in malignant cells. Indeed, in some cases, we could observe in the same tissue section, areas containing normal-looking ducts that displayed weaker BLCAP staining (Fig. 2G, black arrow) than that of adjacent malignant cells (Fig. 2G, red arrow) indicating that expression of BLCAP was increased in malignant cells in comparison to normal tissue. Moderate to strong BLCAP immunoreactivity could also be observed in early lesions, where carcinoma in situ cells showed overexpression of this protein (illustrated in Fig. 2H, grey arrows) in relation to normal adjacent areas (black arrow), and at levels comparable to invasive carcinoma cells (Fig. 2H, red arrow), suggesting that up-regulation of BLCAP, when it takes place, is most likely an early event in breast cancer progression. In order to objectively evaluate this differential BLCAP expression in tumor cells we performed quantitative IHC analysis of BLCAP expression in breast tissues.

### Quantitative Immunohistochemistry Analysis of BLCAP Protein Expression

**Figure 3:**
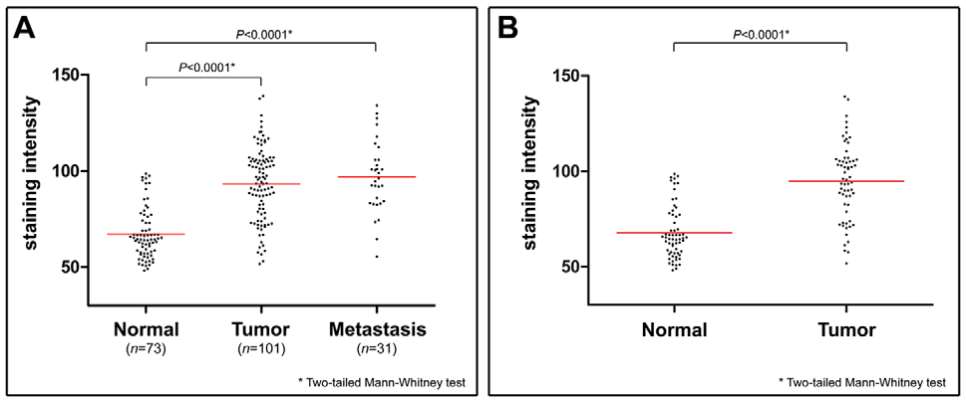
Differential expression of BLCAP in tumor samples. The DCTB 123 patient set, including normal, tumor and lymph node metastasis samples (A), or a subset of matched 62 normal and tumor samples (B) were analyzed by quantitative IHC. Mean intensity scores for each group are indicated by red lines.

Expression of BLCAP in breast tumors was examined, using an automated cellular imaging system (ACIS) (see methods section), by quantitative IHC analysis of FFPE sections of all tissue blocks available from the DCTB 123 patient dataset (Table 1). This sample set consisted of FFPE blocks of matched tissue samples from normal, malignant tissue, and lymph node metastases. For each tissue section, five random, distributed areas (Ø250 µm) of invasive cancer component or normal mammary epithelium, respectively, were chosen for quantification to reduce potential sampling variations. The overall section staining intensity was calculated as the mean value of the five areas. We obtained interpretable, quantifiable staining results for BLCAP in a total of 205 specimens, comprising 73 normal, 101 tumor, and 31 lymph node metastasis tissue samples. Staining intensities were significantly lower in normal samples as compared to tumor samples (Fig. 3A; *P*<0.0001) or lymph node metastases (Fig. 3A; *P*<0.0001). There was no significant difference in BLCAP IHC staining intensity between tumors and lymph node metastases (Fig. 3A; *P* = 0.4971). These data showed that levels of expression of BLCAP are generally higher in malignant cells as compared to normal cells.

**Figure 4:**
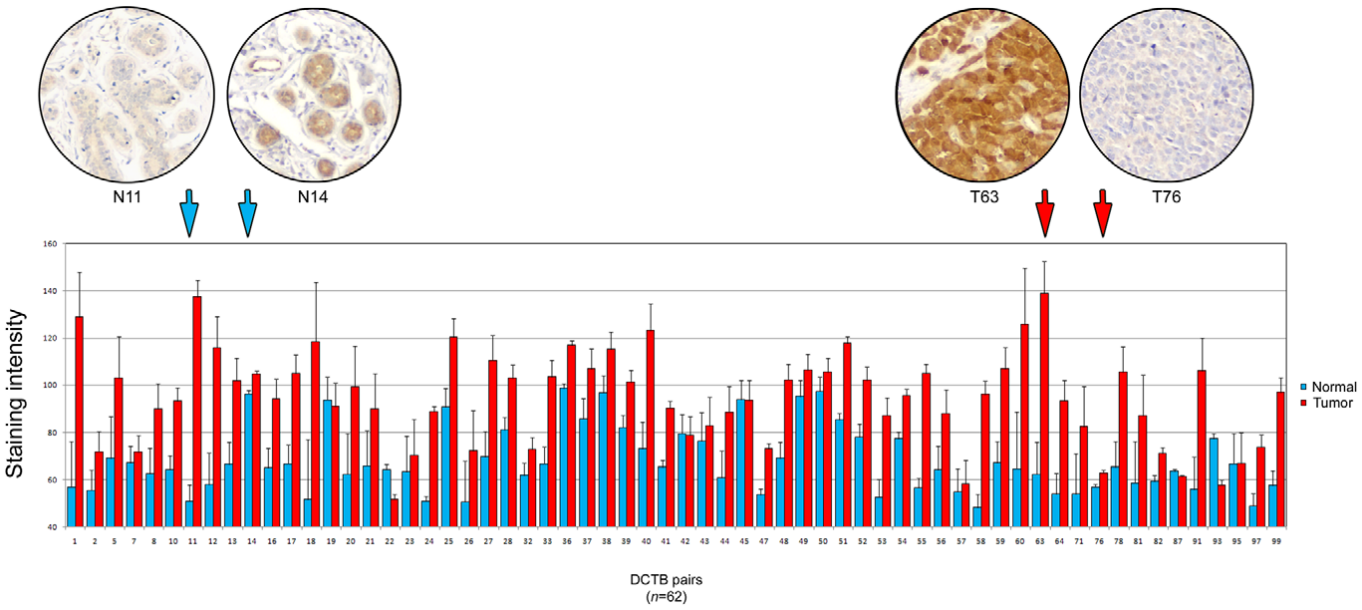
Expression analysis of BLCAP by quantitative IHC of normal specimens (blue bars) and corresponding tumor samples (red bars) of 62 matched cases from the DCTB dataset. Illustrative IHC images are shown for normal and tumor samples with weak immunoreactivity (N11 and T76, respectively), and for normal and tumor samples showing substantial immunoreactivity for BLCAP (N14 and T63, respectively). Magnification 20X.

To determine the proportion of cases showing up-regulation of BLCAP with neoplastic transformation, we performed additional quantitative IHC analysis of only the matched pairs of samples, consisting of normal and corresponding carcinoma, from the DCTB dataset. Of the 123 cases that comprise the DCTB dataset, we identified 62 for which we had well-matched pairs of normal and malignant tissue with concomitant interpretable staining results for BLCAP. We found that the staining intensity, and consequently the expression of BLCAP, varied widely from sample to sample, ranging from non-detectable to strong, both for normal and tumor samples (Fig. 4). In spite of this marked intertumoral heterogeneity, only two cases - DCTB sample pairs 22 and 93, displayed decreased expression levels of BLCAP in tumour tissue as compared to matched normal tissue (Fig. 4). In five cases we found similar expression levels of BLCAP in normal and tumour samples (DCTB pairs 19, 42, 45, 87 and 95), whereas in the 55 remaining cases (88%) we observed increased BLCAP expression in tumour samples relative to the matched normal tissue. In 28 cases (45.2%) the staining intensity for BLCAP was increased in malignant lesions by more than 1.5-fold. Representative IHC images with low and high staining intensity, of normal samples (N11 and N14, respectively), and tumor samples (T76 and T63, respectively), respectively, are presented to illustrate the range of staining intensities (Fig. 4). In spite of the marked intertumoral heterogeneity we discovered, overall levels of expression of BLCAP were significantly higher in malignant samples as compared to matched normal samples (Fig. 3B).

### Analysis of BLCAP Expression in an Independent Sample Set Consisting of 2,197 Breast Tumors: Correlation with the Clinicopathologic Parameters of Tumors

To assess the potential relevance of BLCAP as a breast cancer marker and taking in consideration that having an independent sample dataset is an important feature in the validation of biomarkers, we evaluated the expression of this protein in a breast cancer tissue microarray (TMA) containing 2,197 tumors [41]. We obtained interpretable staining results in 1,899 out of 2,197 (86%) tissue cores arrayed on the TMA. Lack of tumor cells and complete absence of cores were the main reasons for failure to obtain IHC data. Analysis of the IHC staining results from the TMA showed that there was no statistically significant association between BLCAP ACIS TMA score and tumor histological type (Table 2). Moreover, of the other histopathological parameters we assessed, specifically: tumor size and age, nodal status, BRE histologic grade (according to Elston and Ellis) [42], Ki67 index, ER status, PR status, or Her-2/neu status, none showed a statistically significant correlation with BLCAP ACIS TMA score (Table 2). Interestingly, we found that 13.2% of lobular carcinomas present in the TMA had nuclear expression of BLCAP compared to only 5.5% of ductal carcinomas showing that nuclear expression of BLCAP was significantly associated with a specific tumor histological type, invasive lobular carcinoma (*P* = 0.0018 by Fisher’s exact test).

**Table 2:** Clinicopathological correlation of BLCAP expression in 2,197 sample TMA.

	*n*	BLCAP score (median)	*P*
Age (years)			
<60	571	1.67	
60–70	325	1.71	0.115*
>70	391	1.67	
Tumor size (mm)			
≤25	870	1.71	0.100**
>25	634	1.69	
Histologic type			
Ductal	1103	1.71	
Lobular	189	1.64	0.06*
Other	241	1.71	
Histologic grade (BRE)			
Grade 1	351	1.71	
Grade 2	575	1.67	0.152*
Grade 3	508	1.71	
Ki67 index (%)			
<10	126	1.67	
10–20	335	1.67	0.06*
>20	840	1.71	
Lymph node status			
Positive	883	1.67	0.05**
Negative	653	1.74	
Estrogen status			
Positive	1114	1.71	0.217**
Negative	361	1.71	
Progesterone status			
Positive	479	1.69	0.205**
Negative	929	1.71	
HER-2/neu status			
0	1121	1.67	
1	127	1.74	0.07*
2	47	1.71	
3	167	1.74	

* Kruskal-Wallis one way analysis of variance on ranks.

** Mann-Whitney rank-sum test.

### Prognostic Relevance of BLCAP Expression

Analyses of overall survival were performed on the results from the cores present in the TMA for which clinical data and interpretable BLCAP staining were concurrently available (*n* = 1536; corresponding to 526 registered events). Initially, BLCAP was analyzed for prognostic value using the ACIS TMA score as a continuous variable, with univariate hazard ratios calculated by the Cox proportional hazards regression model. Increasing BLCAP overall protein levels were significantly associated with poor survival (*P*<0.01; hazard ratio (HR) = 1.10; [95% confidence interval (95%CI) 1.05–1.15]). Then, to investigate further the prognostic utility of BLCAP, and because a biologically relevant cut-point value for BLCAP protein expression was not known, we followed a standard approach for exploratory analyses of prognostic factors and categorized BLCAP expression levels according to tertiles or median of BLCAP expression. Overall survival analysis for events when patients are dichotomously categorized as having low BLCAP (lower tertile; ACIS III TMA score ≤1.61) or high BLCAP (combined upper two tertiles) expression (HR = 1.19 [95%CI, 1.00–1.43]), showed a modest, but statistically significant, negative association of BLCAP expression with clinical outcome (*P* = 0.0433). Furthermore, multiple pairwise comparison showed that the lowest tertile differed significantly from the highest as evaluated by log-rank test (*P* = 0.017).

When adjusted for potential confounders (age, tumor size, BRE grade, and lymph node status) in a multivariate analysis, using the Cox proportional hazards model, BLCAP expression dichotomized according to low BLCAP (lower tertile; ACIS III TMA score ≤1.61) or high BLCAP (combined upper two tertiles) did not show a statistically significant association of BLCAP expression with clinical outcome (*P* = 0.0661), indicating that BLCAP expression does not have independent overall prognostic value. Also, no significant association was found between BLCAP expression and overall survival (*P* = 0.110), when patients were dichotomized using the score median (ACIS III TMA score 1.71; HR = 1.15 [95%CI 0.97–1.36]).

Analysis of overall survival for specific histopathological types were performed on the results from the invasive ductal carcinoma (IDC) specimens (*n* = 1102; corresponding to 398 registered events), or invasive lobular carcinomas (ILCs) (*n* = 189; corresponding to 65 registered events), as no other histological type had sufficient data to perform this type of analysis. Again, BLCAP was analyzed for prognostic value using the ACIS TMA score as a continuous variable, with univariate hazard ratios calculated by the Cox proportional hazards regression model, showing that BLCAP protein expression levels were not significantly associated with survival in IDCs (*P* = 0.191; hazard ratio (HR) = 0.997; [95% confidence interval (95%CI) 1.05–1.15]) nor in ILCs (*P* = 0.158; hazard ratio (HR) = 0.989; [95% confidence interval (95%CI) 0.975–1.004]).

Given that we previously found that nuclear localization of BLCAP was associated with disease outcome in bladder cancer [13], we also examined the effect of nuclear localization of BLCAP on disease outcome. Survival analysis performed by the Cox proportional hazards model using BLCAP nuclear localization as dichotomous variable (nuclear staining or not) showed no prognostic value (HR = 1.18 [95%CI 0.80–1.74]) of nuclear localization of BLCAP in breast cancer. Since we found that nuclear expression of BLCAP was associated with ILCs (*P* = 0.0018 by Fisher’s exact test), we examined further the effect of nuclear localization of BLCAP on disease outcome specifically in lobular carcinomas. We examined the effect of BLCAP nuclear localization on overall survival in lobular carcinomas using BLCAP nuclear expression as dichotomous variable (nuclear staining or not). A Cox proportional hazards model analysis showed an enhanced hazard of death (HR = 1.84 [95%CI 0.98–3.48]) for the group of women with lobular carcinomas with nuclear BLCAP expression. After adjusting for the influence of several variables (age, tumor size, BRE grade, and lymph node status), BLCAP nuclear presence in lobular carcinomas retained independent, albeit modest, prognostic value (P = 0.042). Although the number of patients in our analysis was limited (*n* = 189; corresponding to 65 events), and the effect on disease outcome was very modest, the trend is similar to what we have observed for bladder cancer, suggesting that BLCAP may have multiple functions, with tissue and cell-specificity.

## Discussion

We have previously reported a number of molecular markers that define particular subpopulations of breast epithelial cells [14], [16], [18], [43]; although we could demonstrate differential expression of some of these markers in breast tumors, and thus document their potential to serve as candidate biomarkers, ultimately, their clinical usefulness may rather lay as part of a multi-marker panel of proteins that can be used for diagnosis and/or prognosis of breast lesions. While it is reasonable to assume that using a panel of biomarkers may have enhanced diagnostic/prognostic value over a single marker [13], [32]–[39], [44], the added methodological complexity of multiple markers makes it necessary to optimize and select combinations of markers based on their overall diagnostic utility. Consequently, in order to maximize the probability of success of a multiplexing strategy, it is desirable to have a large pool of candidate protein biomarkers to identify and validate the biomarker combination with the highest possible clinical value. With this in mind we evaluated the potential of BLCAP, a marker we previously identified in urothelial carcinomas, as possible breast cancer biomarker and prognostic factor. We investigated the expression and localization of BLCAP in breast tissue, normal as well as malignant, by IHC, analyzing the distribution of the immunostaining, and its intensity. We found that BLCAP was expressed at low levels in normal breast epithelial cells (Figs. 2B and 3A), with weak cytoplasmic staining and rare nuclear staining. The generally low levels of immunostaining we found in normal breast epithelial cells, as compared to what we previously reported for urothelial cells [13], are consistent with gene expression data (HG-U133A array platform) for *BLCAP* that shows that expression of *BLCAP* mRNA is up-regulated in normal bladder and down-regulated in normal breast tissue [45]. But also the staining pattern for BLCAP was clearly different to what we had previously observed for normal bladder tissue.

Even though immunoreactivity for BLCAP in normal breast tissue varied greatly from sample to sample (Fig. 4, compare N11 with N14), we did not observe pronounced nuclear localization of BLCAP as it was the case for bladder tissue. In addition to the differences in cellular expression and localization of BLCAP in normal tissue, we also found that the pattern of BLCAP expression in malignant tissue was very different in breast and in bladder tumors. We previously reported a dual behavior of BLCAP in bladder tumors; we observed down-regulation of BLCAP protein associated with progression (up to 51.2% of invasive tumors had weak or undetectable expression), and overexpression of BLCAP only in a minor percentage of tumors (up to 20% of all cases, irrespective of tumor stage/grade) [13]. By contrast, breast cancer specimens with no detectable immunoreactivity for BLCAP, constituted only a minor part of the total number of samples (8 out of 101 samples). In general, tumor samples showed increased expression of BLCAP as compared to normal samples (Figs. 3 and 4). Indeed, we found that 28 cases out of 62 (45.2%), for which we had matched pairs of normal and malignant tissue with interpretable staining results for BLCAP, displayed an increase in BLCAP expression of more than 1.5-fold in breast tumor cells compared to normal cells (Fig. 4). Overall, these data are indicative of a cell and tissue-specific expression pattern for BLCAP. But it also raises one vital question, since at first sight these observations are incongruent with the idea that BLCAP may have a tumor-suppressor function [20], [22]–[25]. Ectopic overexpression of this protein in cell lines inhibits cell growth, and induces apoptosis [21], [24], [25], yet, out of the 62 well-matched pairs of normal and malignant tissue we had available, 60 cases displayed similar or increased expression levels of BLCAP.

One possible explanation is that in addition to displaying tissue-specific expression patterns, BLCAP also has tissue-specific functions. Consequently, it is conceivable that the tumor suppressive effect of BLCAP is not functional in mammary epithelial cells. However, it is more likely that although the relative expression levels of BLCAP are increased in breast tumor cells, the absolute levels of expression remain below the threshold for cytotoxicity. Our inability to detect BLCAP in silver stained 2D protein gels of whole tissue lysates, even in samples showing strong immunoreactivity for BLCAP by IHC (see for example T63 in Fig. 1D and Fig. S1; and IHC panel in Fig. 4), showed that in all cases examined, expression of this protein was below the sensitivity limit of the assay, suggesting that in the samples that displayed augmented relative levels of BLCAP by IHC, the absolute levels of this protein remained very low. These data are consistent with the observation that ectopic expression of BLCAP protein at very high, and hence non-physiological, levels is deleterious for cells [21, 24, and data not shown] and would explain why we observed frequent up-regulation of a presumed tumor suppressor in breast carcinomas.

We showed previously that in urothelial carcinomas, in most cases, BLCAP protein expression was lost with tumor progression. At the same time, we also showed that BLCAP was overexpressed in 20% of the cases, independently of the clinicopathological parameters examined, and that overexpression correlated with poor survival [13]. Here we showed that, in breast cancer, there was no statistically significant association between BLCAP staining score and histological type (Table 2). Moreover, none of the other histopathological parameters we assessed, specifically: tumor size and age, nodal status or BRE histologic grade (according to Elston and Ellis) [42], ER status, PR status, or Her-2/neu status showed any statistically significant correlation with BLCAP staining score (Table 2). We also found that BLCAP expression did not have independent prognostic value. Taken together these data indicate that BLCAP may have multiple, tissue-specific functions in cancer cells, with one or more of those function(s) conferring a more aggressive behavior to cancer cells. This function(s) are likely to be associated with nuclear presence [13], since we found that nuclear expression of BLCAP was associated with poor outcome (HR = 1.84 [95%CI 0.98–3.48]) for women with lobular carcinomas displaying nuclear BLCAP expression, even after adjusting for the influence of several variables (age, tumor size, BRE grade, ER status, and nodal status)(*P* = 0.042). Although the number of patients in our analysis was limited (*n* = 189; corresponding to 65 events), and the effect on disease outcome was very modest, the trend is similar to what we have observed for bladder cancer.

In conclusion, we found that expression of BLCAP was up-regulated in a substantial proportion of breast tumors indicating that BLCAP may be of value as a biomarker for breast cancer. These data together with the results we reported for the behavior of this protein in bladder cancer underline the need for further research into a possible multiple effect of BLCAP in cancer cells, on the one hand having a tumor suppressor role, and on the other hand leading to poor disease outcome. Dissecting the various regulatory networks and pathways controlling BLCAP expression and localization and determining its function(s), in particular that associated with nuclear expression, might provide a clearer understanding of the roles this protein plays in carcinogenesis, a fact that will enhance its usefulness as a cancer biomarker, alone or in combination with others.

## Materials and Methods

### Sample Collection, Handling, and Ethics Statement

Tissue samples from clinical high-risk patients (high-risk definition according to the Danish Breast Cooperative Group; www.dbcg.dk) that underwent mastectomy between 2003 and 2008 were provided by the Department of Pathology at the Copenhagen University Hospital. One hundred twenty three women with primary, operable, high-risk invasive breast cancer were selected for this prospective study (Danish Center for Translational Breast Cancer Research - DCTB patient numbers 1 to 123). Written informed consent was received from all participants. Clinicopathological data for patients are given in Table 1. Patients had had no previous surgery of the breast and had not received preoperative treatment. Patients underwent mastectomy, which included axillary dissection in those cases with positive sentinel nodes. Following surgery, fresh tissue samples were divided into various pieces and immediately processed. On all cases no more than 30 min elapsed from tissue acquisition to processing; all samples were kept on ice in the intervening time. Matched non-malignant tissue and lymph node metastases were also collected whenever possible. The project was approved (KF 01-069/03) by the Copenhagen and Frederiksberg regional division of the Danish National Committee on Biomedical Research Ethics. Normal tissue biopsies were also procured from discarded anonymous excess tissue from reduction mammoplasties (Erichsens Privathospital, Denmark).

### Immunohistochemical (IHC) Analysis and Tissue Microarrays

Five-µm sections were cut from the tissue blocks and mounted on Super Frost Plus slides (Menzel-Gläser, Braunschweig, Germany), baked at 60°C for 60 min, deparaffinized, and rehydrated through graded alcohol rinses. Heat induced antigen retrieval was performed by immersing slides immersing the slides in Tris/EDTA pH 9.0 buffer (10 mM Tris, 1 mM EDTA) and microwaving in a 750 W microwave oven for 10 min. The slides were then cooled at room temperature for 20 min and rinsed abundantly in tap water. Non-specific staining of slides was blocked (10% normal goat serum in PBS buffer) for 15 min, and endogenous peroxidase activity quenched using 0.3% H_2_O_2_ in methanol for 30 min. Antigen presence was visualized by incubation for 1 h with the primary antibody (anti-BLCAP antibody, EP023514; used at 1∶250, Eurogentec), followed by detection with a suitable species-matched secondary antibody conjugated to a peroxidase complex for 30 min (Envision+ poly-HRP system; DAKO, Denmark). Color development was done using DAB+ Chromogen (DAKO, Denmark). Slides were counterstained with hematoxylin. Standardization of the incubation and development times allowed accurate comparisons in all cases. The BLCAP rabbit polyclonal antibody was immunoaffinity purified against the immunizing peptide prior to use. Normal rabbit serum instead of primary antibody was generally used as a negative control. Construction of the breast prognosis tissue microarray (TMA) has been described in detail elsewhere [41].

### Quantitative Assessment of Immunohistochemistry (IHC) Staining and Data Analysis

An automated cellular imaging system, ACIS™ III (DAKO, Denmark), was used to digitize and quantify IHC staining intensity of tissue sections. The ACIS system is capable of simultaneously detecting levels of hue (color), saturation (density) and luminosity (darkness). By using the ACIS proprietary software, users can define threshold values for each of these parameters thus allowing the system to separately recognize brown pixels (positive immunostaining) and blue pixels (hematoxylin counterstain). For each tissue section, five distributed representative areas (Ø250 µm) of similar grade were defined and staining intensity values determined for each area. The overall section staining intensity was calculated as the mean value of the five areas and is referred to as “staining intensity”. For analysis of TMAs, the ACIS™ III system was used to derive a score for each core. The digital images from scanned TMA sections were submitted to analysis by the TMA proprietary software module that is part of the ACIS™ III system. A staining score which is a function of staining intensity and the percentage of cells showing immunoreactivity was generated in this manner for each core and is referred to as “ACIS TMA score”.

### 2D PAGE and 2D Western blotting

2D PAGE and 2D Western blotting were performed as described previously [43].

### Transient Expression of BLCAP cDNA in COS-1 Cells

The full length BLCAP cDNA containing the entire coding region was subcloned into the mammalian expression vector pZeoSV2 (Life Technologies, USA) and transiently transfected into COS-1 cells labelled with ^35^S-methionine using the Lipofectamine® Transfection Reagent (Life Technology, USA) according to the manufacturer’s descriptions.

### Protein Spot Handling and Mass Spectrometry Analysis

Mass spectrometry analysis of spots of interest excised from the gel was performed as previously described [16)] “In- gel” digestion was accomplished either by using sequencing grade trypsin or chymotrypsin purchased from (Roche, USA and Cambio, UK) correspondingly in accordance to manufactory protocol. MALDI TOF/TOF mass spectrometry was performed for peptide mass fingerprinting (PMF) as described previously [16] by using Ultraflex ™ III 200 time-of-flight mass spectrometer (Bruker Daltonik, Germany) equipped with a Smart beam™ laser and a LIFT-TOF/TOF unit. Data acquisition and data processing were performed by the Flex Control 3.0 and Flex Analysis 3.0 software (Bruker Daltonik, Germany). All of the spectra were obtained using reflector positive mode with an acceleration voltage of 25 kV, reflector voltage of 26.38 kV and detection suppressed up to 450 Da. A total of 2000 shots in steps of 200 shots were added to one spectrum in the mass range of m/z 600–4000 using peak detection algorithm: SNAP (Sort Neaten Assign and Place); S/N threshold: 3 and Quality Factor Threshold: 50. Automatic external calibration was performed using a peptide mixture purchased from (Bruker Daltonik, Germany) followed by internal calibration as described previously [16].

### Statistical Analysis

The groups classified by IHC ACIS scores were compared using the Kruskal-Wallis analysis of variance, and Mann-Whitney tests. Survival analysis was performed by the Cox proportional hazards model using the ACIS III™ intensity score as a continuous variable or by the Kaplan-Meier method, using various cutpoints to dichotomize the intensity scores, with significance evaluated using the log-rank test. To correct for the influence of several variables (patient and tumor characteristics) we performed multivariate analysis using the Cox proportional hazards model. In all analyses a two-sided significance level of0.05 was used. All statistical analysis were conducted using SAS statistical software (version. 9.3; SAS Institute, Inc., Cary, NC).Due to missing values the number of subjects varies between the examined variables.

## Supporting Information

Figure S1 **Silver stained 2D-PAGE (IEF) gels and respective 2D Western blots of representative primary breast tumors that showed both (A) strong (T63) and (B) weak (T76) immunoreactivity for BLCAP by IHC.** The position of the BLCAP protein in the 2D-PAGE gels, inferred from the corresponding 2D Western blot, is indicated. The positions of 14-3-3 proteins are indicated as references. Reversible staining of blot transfer membranes with Ponceau S was performed to monitor the extent of transfer and confirm comparable protein lysate loading, as well accurately position the detected spots with respect to the silver stained gels. 2D Western blot analysis showed that signal intensities for BLCAP paralleled the IHC results, with a sample that showed strong immunoreactivity in IHC (T63) also showing a strong signal (A), whereas T76, which displayed weak immunoreactivity in IHC, had a weak signal in immunoblotting (B), respectively.
(TIF)

Figure S2 **Identification of BLCAP by mass spectrometry.** The positions of protein spots on the gel were determined by superimposition with corresponding ^35^S-autograph. Spot of interest was excised from the dry 2D gel containing separated COS-1 cells transfected with pZeoSV2– BLCAP construct. A novel protein spot of MW 10 kDa and pI 6.2 present in COS-1 cells transiently transfected with pZeoSV2-BLCAP but not in control cells (compare Fig. 1A with 1B, black arrows) was analyzed by mass spectrometry confirming the identity of the protein as BLCAP.
(TIF)

## References

[1] Celis JE, Gromov P (2003) Proteomics in translational cancer research: toward an integrated approach. Cancer Cell 3: 9–15. 12559171 10.1016/s1535-6108(02)00242-8

[2] Celis JE, Gromov P, Gromova I, Moreira J M, Cabezón T, et al (2003) Integrating Proteomic and Functional Genomic Technologies in Discovery-driven Translational Breast Cancer Research. Mol Cell Proteomics 2: 369–377. 12832461 10.1074/mcp.R300007-MCP200

[3] Celis JE, Gromova I, Moreira JM, Cabezon T, Gromov P (2004) Impact of proteomics on bladder cancer research. Pharmacogenomics 5: 381–394. 15165174 10.1517/14622416.5.4.381

[4] Celis JE, Moreira JM, Gromova I, Cabezón T, Ralfkiaer U, et al (2005) Towards discovery-driven translational research in breast cancer. FEBS J 272: 2–15. 15634327 10.1111/j.1432-1033.2004.04418.x

[5] Gromov P, Moreira JM, Gromova I, Celis JE (2008) Proteomic strategies in bladder cancer: From tissue to fluid and back. Proteomics Clin Appl 2: 974–988. 21136898 10.1002/prca.200780163

[6] Østergaard M, Rasmussen HH, Nielsen HV, Vorum H, Ørntoft TF, et al (1997) Proteome profiling of bladder squamous cell carcinomas: identification of markers that define their degree of differentiation. Cancer Res. 57: 4111–4117. 9307301

[7] Gromova I, Gromov P, Wolf H, Celis JE (1998) Protein abundancy and mRNA levels of the adipocyte type fatty acid binding protein correlate in non-invasive and invasive bladder transitional cell carcinomas. Int J Oncol 13: 379–383. 9664136 10.3892/ijo.13.2.379

[8] Østergaard M, Wolf H, Ørntoft TF, Celis JE (1999) Psoriasin (S100A7): a putative urinary marker for the follow-up of patients with bladder squamous cell carcinomas. Electrophoresis 20: 349–354. 10197442 10.1002/(SICI)1522-2683(19990201)20:2<349::AID-ELPS349>3.0.CO;2-B

[9] Moreira JM, Gromov P, Celis JE (2004) Expression of the tumor suppressor protein 14-3-3σ is down-regulated in invasive transitional cell carcinomas of the urinary bladder undergoing epithelial-to-mesenchymal transition. Mol Cell Proteomics 3: 410–419. 14736829 10.1074/mcp.M300134-MCP200

[10] Celis JE, Celis P, Palsdottir H, Østergaard M, Gromov P, et al (2002) Proteomic strategies to reveal tumor heterogeneity among urothelial papillomas. Mol Cell Proteomics 1: 269–279. 12096109 10.1074/mcp.m100031-mcp200

[11] Gromova I, Gromov P, Celis JE (2002) Bc10: A novel human bladder cancer-associated protein with a conserved genomic structure downregulated in invasive cancer. Int J Cancer 98: 539–546. 11920613 10.1002/ijc.10244

[12] Ohlsson G, Moreira JMA, Gromov P, Sauter G, Celis JE (2005) Loss of expression of the adipocyte-type fatty acid-binding protein (A-FABP) is associated with progression of human urothelial carcinomas. Mol Cell Proteomics 4: 570–581. 15734831 10.1074/mcp.M500017-MCP200

[13] Moreira JM, Ohlsson G, Gromov P, Simon R, Sauter G, et al (2010) Bladder cancer-associated protein, a potential prognostic biomarker in human bladder cancer. Mol Cell Proteomics 9: 161–177. 19783793 10.1074/mcp.M900294-MCP200 PMC2808262

[14] Celis JE, Gromova I, Cabezón T, Gromov P, Shen T, et al (2007) Identification of a subset of breast carcinomas characterized by expression of cytokeratin 15: relationship between CK15+ progenitor/amplified cells and pre-malignant lesions and invasive disease. Mol Oncol 1: 321–349. 19383306 10.1016/j.molonc.2007.09.004 PMC5543867

[15] Celis JE, Cabezón T, Moreira JM, Gromov P, Gromova I, et al (2009) Molecular characterization of apocrine carcinoma of the breast: Validation of an apocrine protein signature in a well-defined cohort. Mol Oncol 3: 220–237. 19393583 10.1016/j.molonc.2009.01.005 PMC5527852

[16] Gromov P, Gromova I, Friis E, Timmermans-Wielenga V, Rank F, et al (2010) Proteomic profiling of mammary carcinomas identifies C7orf24, a gamma-glutamyl cyclotransferase, as a potential cancer biomarker. J Proteome Res. 9: 3941–3953. 10.1021/pr100160u 20527979

[17] Celis JE, Gromov, P. Cabezón T, Moreira JM, Ambartsumian N, et al (2004) Proteomic characterization of the interstitial fluid perfusing the breast tumor microenvironment: a novel resource for biomarker and therapeutic target discovery. Mol Cell Proteomics 3 327–344. 14754989 10.1074/mcp.M400009-MCP200

[18] Gromov P, Gromova I, Bunkenborg J, Cabezón T, Moreira JM, et al (2010) Up-regulated proteins in the fluid bathing the tumour cell microenvironment as potential serological markers for early detection of cancer of the breast. Mol Oncol 4: 65–89. 20005186 10.1016/j.molonc.2009.11.003 PMC5527961

[19] Zuo Z, Zhao M, Liu J, Gao G, Wu X (2006) Functional analysis of bladder cancer-related protein gene: a putative cervical cancer tumor suppressor gene in cervical carcinoma. Tumour Biol 27: 221–226. 16675915 10.1159/000093057

[20] Rae FK, Stephenson SA, Nicol DL, Clements JA (2000) Novel association of a diverse range of genes with renal cell carcinoma as identified by differential display. Int J Cancer 88: 726–732. 11072240 10.1002/1097-0215(20001201)88:5<726::aid-ijc7>3.0.co;2-h

[21] Yao J, Duan L, Fan M, Yuan J, Wu X (2007) Overexpression of BLCAP induces S phase arrest and apoptosis independent of p53 and NF-kappaB in human tongue carcinoma: BLCAP overexpression induces S phase arrest and apoptosis. Mol Cell Biochem 297: 81–92. 17031575 10.1007/s11010-006-9332-2

[22] Su HC, Zhao YH, Fan DG, Fan QY, Zhang P, et al (2003) Relationship between expression of BLCAP protein and malignancy of osteosarcoma. Xi Bao Yu Fen Zi Mian Yi Xue Za Zhi 19: 465–466. 15169658

[23] Daino K, Ugolin N, Altmeyer-Morel S, Guilly MN, Chevillard S (2009) Gene expression profiling of alpha-radiation-induced rat osteosarcomas: identification of dysregulated genes involved in radiation-induced tumorigenesis of bone. Int J Cancer 125: 612–620. 19444910 10.1002/ijc.24392

[24] Fan DG, Zhao F, Ding Y, Wu MM, Fan QY, et al (2011) BLCAP induces apoptosis in human Ewing’s sarcoma cells. Exp Biol Med 236: 1030–1035. 10.1258/ebm.2011.010315 21844121

[25] Zuo ZH, Zhao M, Liu J, Wei Y, Wu XX (2006) Inhibitory effect of bladder cancer related protein gene on HeLa cell proliferation. Ai Zheng 25: 811–817. 16831269

[26] Galeano F, Leroy A, Rossetti C, Gromova I, Gautier P, et al (2010) Human BLCAP transcript: new editing events in normal and cancerous tissues. Int J Cancer 127: 127–137. 19908260 10.1002/ijc.25022 PMC2958456

[27] Levanon EY, Hallegger M, Kinar Y, Shemesh R, Djinovic-Carugo K, et al (2005) Evolutionarily conserved human targets of adenosine to inosine RNA editing. Nucleic Acids Res 33: 1162–1168. 15731336 10.1093/nar/gki239 PMC549564

[28] Clutterbuck DR, Leroy A, O’Connell MA, Semple CA (2005) A bioinformatic screen for novel A-I RNA editing sites reveals recoding editing in BC10. Bioinformatics 21: 2590–2595. 15797904 10.1093/bioinformatics/bti411

[29] Riedmann EM, Schopoff S, Hartner JC, Jantsch MF (2008) Specificity of ADAR-mediated RNA editing in newly identified targets. RNA 14: 1110–1118. 18430892 10.1261/rna.923308 PMC2390793

[30] Allred DC (2011) Molecular Biomarkers of Risk in Premalignancy and Breast Cancer Prevention. Cancer Prev Res 4: 1947–1952. 10.1158/1940-6207.CAPR-11-0478 22144468

[31] Cole K, Tabernero M, Anderson KS (2011) Biologic characteristics of premalignant breast disease. Cancer Biomark 9: 177–192. 10.3233/CBM-2011-0187 PMC3432637 22112476

[32] Misek DE, Kim EH (2011) Protein biomarkers for the early detection of breast cancer. Int J Proteomics. 2011: 343582. 10.1155/2011/343582 PMC3195294 22084684

[33] Edgell T, Martin-Roussety G, Barker G, Autelitano DJ, Allen D, et al (2010) Phase II biomarker trial of a multimarker diagnostic for ovarian cancer. J Cancer Res Clin Oncol 136: 1079–88. 20082099 10.1007/s00432-009-0755-5 PMC2874491

[34] Adam BL, Qu Y, Davis JW, Ward MD, Clements MA, et al (2002) Serum protein fingerprinting coupled with a pattern-matching algorithm distinguishes prostate cancer from benign prostate hyperplasia and healthy men. Cancer Res 62: 3609–14. 12097261

[35] Kashani-Sabet M, Venna S, Nosrati M, Rangel J, Sucker A, et al (2009) A multimarker prognostic assay for primary cutaneous melanoma. Clin Cancer Res 15: 6987–92. 19887476 10.1158/1078-0432.CCR-09-1777 PMC2784204

[36] Birkhahn M, Mitra AP, Cote RJ (2007) Molecular markers for bladder cancer: the road to a multimarker approach. Expert Rev Anticancer Ther 7: 1717–27. 18062746 10.1586/14737140.7.12.1717

[37] Jain KK (2008) Innovations, challenges and future prospects of oncoproteomics. Mol Oncol 2: 153–60. 19383334 10.1016/j.molonc.2008.05.003 PMC5527761

[38] Bichsel VE, Liotta LA, Petricoin EF 3rd (2001) Cancer proteomics: from biomarker discovery to signal pathway profiling. Cancer J 7: 69–78. 11269650

[39] Whiteley GR (2006) Proteomic patterns for cancer diagnosis–promise and challenges. Mol Biosyst 2: 358–63. 16880955 10.1039/b607260g

[40] Pepe MS, Etzioni R, Feng Z, Potter JD, Thompson ML, et al (2001) Phases of biomarker development for early detection of cancer. J Natl Cancer Inst 93: 1054–1061. 11459866 10.1093/jnci/93.14.1054

[41] Al-Kuraya K, Schraml P, Torhorst J, Tapia C, Zaharieva B, et al (2004) Prognostic relevance of gene amplifications and coamplifications in breast cancer. Cancer Res 64: 8534–8540. 15574759 10.1158/0008-5472.CAN-04-1945

[42] Elston CW, Ellis IO (1991) Pathological prognostic factors in breast cancer. I. The value of histological grade in breast cancer: experience from a large study with long-term follow-up. Histopathology 19: 403–10. 1757079 10.1111/j.1365-2559.1991.tb00229.x

[43] Moreira JM, Cabezón T, Gromova I, Gromov P, Timmermans-Wielenga V, et al (2010) Tissue proteomics of the human mammary gland: towards an abridged definition of the molecular phenotypes underlying epithelial normalcy. Mol Oncol 4: 539–361. 21036680 10.1016/j.molonc.2010.09.005 PMC5527921

[44] Zethelius B, Berglund L, Sundstrom J, Ingelsson E, Basu S, et al (2008) Use of multiple biomarkers to improve the prediction of death from cardiovascular causes. N Engl J Med 358: 2107–2116. 18480203 10.1056/NEJMoa0707064

[45] Lukk M, Kapushesky M, Nikkilä J, Parkinson H, Goncalves A, et al (2010) A global map of human gene expression. Nature Biotechnology 28: 322–24. 10.1038/nbt0410-322 PMC2974261 20379172

