# Supplementary figures and images for: Immunoexpression Analysis and Prognostic Value of BLCAP in Breast Cancer
Source: PLoS One. 2012 Sep 25;7(9):e45967. doi: 10.1371/journal.pone.0045967 (PMC3458104; doi:10.1371/journal.pone.0045967)

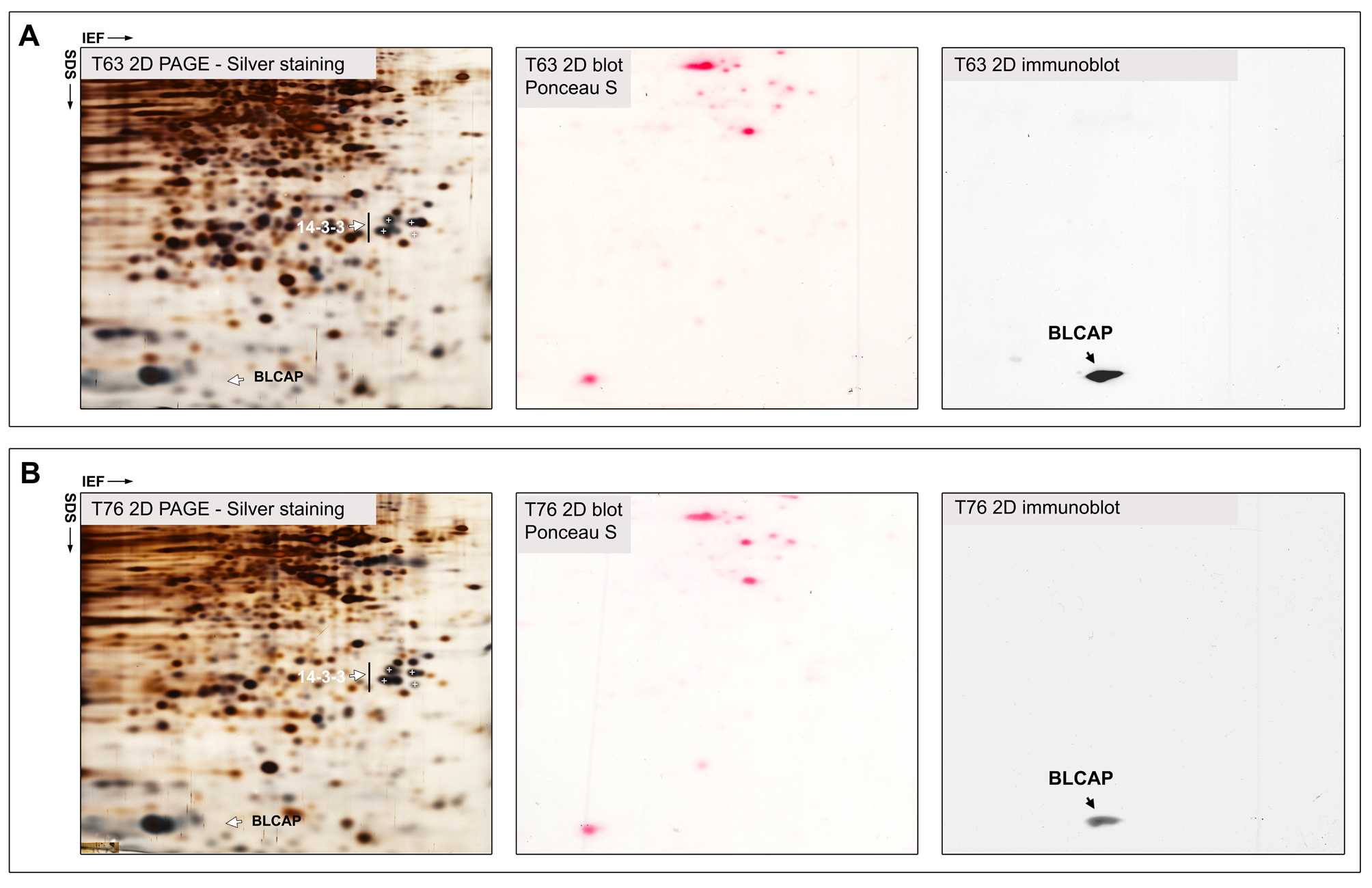

Supplement: Figure S1 — Silver stained 2D-PAGE (IEF) gels and respective 2D Western blots of representative primary breast tumors that showed both (A) strong (T63) and (B) weak (T76) immunoreactivity for BLCAP by IHC. The position of the BLCAP protein in the 2D-PAGE gels, inferred from the corresponding 2D Western blot, is indicated. The positions of 14-3-3 proteins are indicated as references. Reversible staining of blot transfer membranes with Ponceau S was performed to monitor the extent of transfer and confirm comparable protein lysate loading, as well accurately position the detected spots with respect to the silver stained gels. 2D Western blot analysis showed that signal intensities for BLCAP paralleled the IHC results, with a sample that showed strong immunoreactivity in IHC (T63) also showing a strong signal (A), whereas T76, which displayed weak immunoreactivity in IHC, had a weak signal in immunoblotting (B), respectively. (TIF) [file pone.0045967.s001.tif]

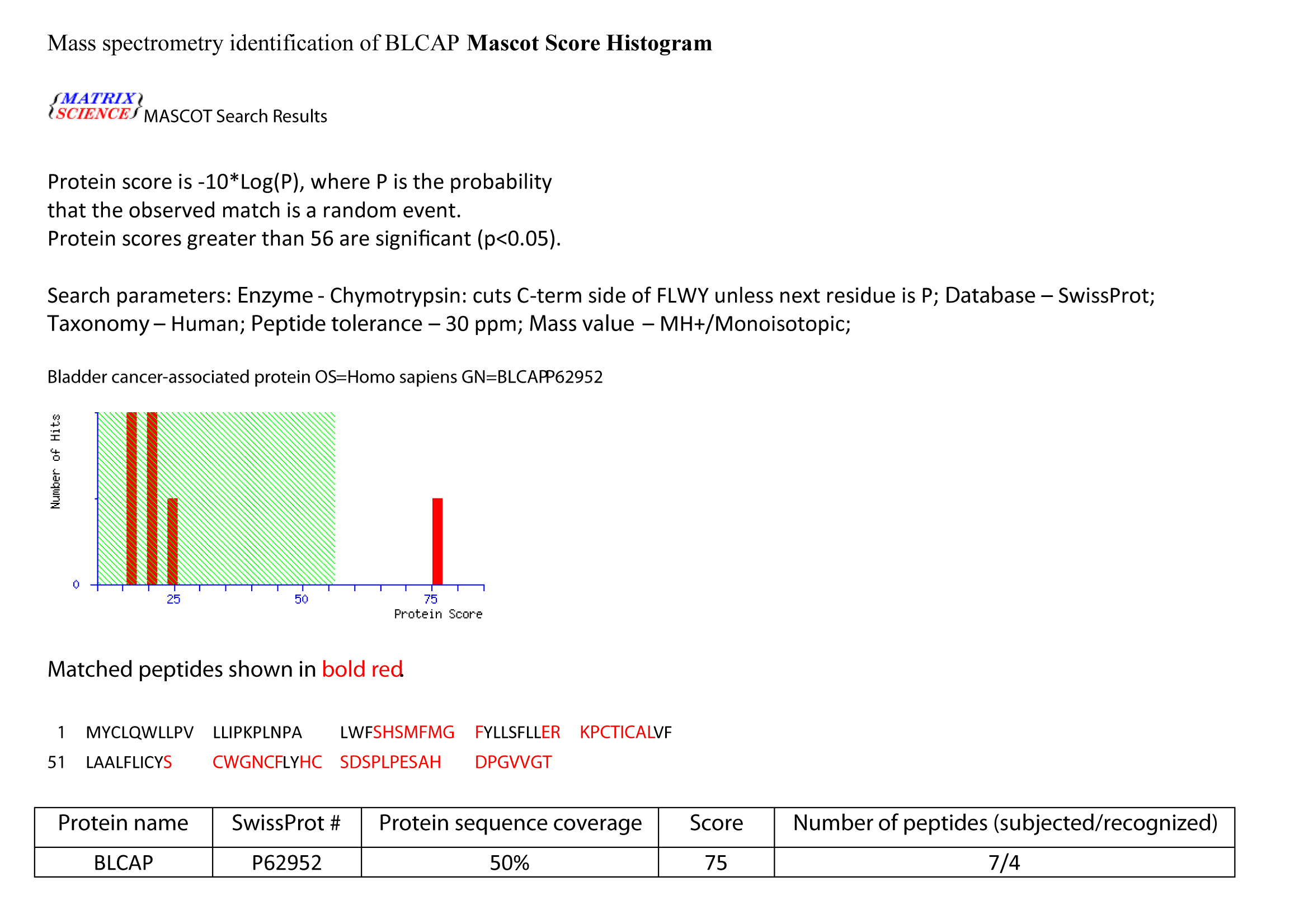

Supplement: Figure S2 — Identification of BLCAP by mass spectrometry. The positions of protein spots on the gel were determined by superimposition with corresponding 35S-autograph. Spot of interest was excised from the dry 2D gel containing separated COS-1 cells transfected with pZeoSV2– BLCAP construct. A novel protein spot of MW 10 kDa and pI 6.2 present in COS-1 cells transiently transfected with pZeoSV2-BLCAP but not in control cells (compare Fig. 1A with 1B, black arrows) was analyzed by mass spectrometry confirming the identity of the protein as BLCAP. (TIF) [file pone.0045967.s002.tif]
